# Effect of Specialized Psychiatric Assessment and Precision Diagnosis on Pharmacotherapy in Adults with Intellectual Disability

**DOI:** 10.3390/jcm15020489

**Published:** 2026-01-08

**Authors:** Marta Basaldella, Michele Rossi, Marco Garzitto, Roberta Ruffilli, Carlo Francescutti, Shoumitro Deb, Marco Colizzi, Marco O. Bertelli

**Affiliations:** 1 Unit of Psychiatry, Department of Medicine (DAME), University of Udine, 33100 Udine, Italy; basaldella.marta@spes.uniud.it (M.B.); marco.colizzi@uniud.it (M.C.); 2 Azienda Sanitaria Friuli Occidentale (ASFO), 33170 Pordenone, Italy; 3 CREA—Research and Clinical Centre, San Sebastiano Foundation, Misericordia di Firenze, 50100 Florence, Italy; mrossi@crea-sansebastiano.org; 4 Department of Brain Sciences, Imperial College London, London SW7 2AZ, UK; s.deb@imperial.ac.uk; 5 Department of Psychosis Studies, Institute of Psychiatry, Psychology and Neuroscience, King’s College London, London SE5 8AF, UK

**Keywords:** neurodevelopmental disorders, autism spectrum disorder, dual diagnosis, diagnostic overshadowing, polypharmacy, medication management

## Abstract

**Background/Objectives**: Adults with intellectual disability (ID) experience high rates of psychiatric comorbidity but often face diagnostic challenges and treatment barriers, leading to inappropriate psychotropic medication use. This study examined the extent to which specialized psychiatric assessment and improved diagnostic accuracy had an impact on medication management and clinical outcomes in adults with ID and co-occurring psychiatric disorders. **Methods**: This observational retrospective study analyzed medical records from 25 adults with ID who underwent specialized psychiatric assessment at a community-based service in Italy between January 2023 and January 2024. Psychopathological diagnoses were established according to Diagnostic Manual—Intellectual Disability, Second Edition (DM-ID2) criteria, based on clinical observation and a comprehensive assessment using validated instruments. Clinical outcomes were assessed using a psychometric tool encompassing multiple psychopathological and behavioral dimensions. Data on psychotropic prescriptions and side effects were also collected. Non-parametric analyses were performed, with significance set at α = 0.05. **Results:** The proportion of patients with a psychiatric diagnosis increased from 32% to 96% after specialized assessment (*p* < 0.001), with notable rises in depressive (0% to 32%), bipolar (8% to 36%), anxiety (4% to 24%), and impulse control (0% to 16%) disorders. First-generation antipsychotic prescriptions decreased (from 36% to 8%, *p* = 0.023), while antidepressant use increased (from 12% to 52%, *p* = 0.004). The mean number of side effects per patient declined from 1.6 to 0.5 (*p* < 0.001), particularly the elevated prolactin level and psychomotor retardation. Significant improvements were observed in symptom intensity and frequency across multiple domains, including aggression, mood disturbances, and compulsions (*p* < 0.001). **Conclusions**: In this single-center retrospective study, specialized psychiatric assessment was associated with improved diagnostic accuracy, medication management, and clinical outcomes in adults with ID. The increase in psychiatric diagnoses likely reflects improved identification, addressing key challenges in precision diagnosis for people with neurodevelopmental disorders. Although the overall number of prescribed medications remained stable, optimization of treatment regimens reduced first-generation antipsychotic use and related adverse effects. These findings indicates that access to specialized assessment and precision diagnosis could improve psychopharmacological interventions and outcomes for this vulnerable population, but larger, multi-center and longer-term studies are needed to confirm these results.

## 1. Introduction

Adults with intellectual disability (ID), particularly those with co-occurring autism spectrum disorder (ASD), represent one of the most psychiatrically vulnerable populations in healthcare, yet they remain among the most underserved and misunderstood [1].

This population experiences psychiatric comorbidity rates of 30–70% [2,3,4], nearly three times higher than the 10–20% prevalence observed in the general population [5,6]. This higher risk may be related to a number of factors, including atypical neurodevelopmental trajectories affecting brain circuits critical for emotional regulation and social cognition, such as the amygdala and prefrontal cortex [7]. Despite this elevated risk, accurate diagnosis and treatment of psychiatric disorders in this population remain a significant challenge [8].

Distinctive features associated with neurodevelopmental disorders complicate the establishment of appropriate psychiatric diagnoses by clinicians lacking specialized skills and training. These include cognitive and communication difficulties, atypical expression of psychological distress, developmental peculiarities, neuroautonomic vulnerability, reduced stress tolerance, and difficulties processing sensory input [9,10]. In addition, the identification of psychiatric disorders can be influenced by how carers and clinicians conceptualize the mental suffering of these individuals. Behaviors or emotional changes that may actually reflect manifestations of untreated psychiatric disorders, underlying medical conditions, responses to environmental stressors, or sensory processing difficulties are in fact often misinterpreted either as behavioral disturbances requiring sedation or as intrinsic features of the neurodevelopmental condition itself [10,11]. The difficulty of distinguishing the manifestation of neurodevelopmental disorders from those arising from co-occurring psychiatric disorders is known as diagnostic overshadowing. This phenomenon extends beyond the mere under-recognition of functional impairments or subjective distress; it also encompasses the misattribution of psychopathological symptoms and their clustering into recognizable syndromes, thus hindering access to appropriate mental healthcare [10,11,12].

This systematic bias leads to a troubling pattern of inappropriate psychotropic medication use. Previous studies have demonstrated that a large proportion of individuals with ID and/or ASD receive psychotropic medication in the absence of a clear psychiatric diagnosis, often to manage problem behaviors rather than specific psychopathological syndromes [4,13,14]. Recent studies reveal alarming prescribing patterns: up to 50% of adults with ID receive antipsychotic medications, often in the absence of psychotic disorders, with polypharmacy rates reaching 30–40% [15,16]. The comorbidity of severe ID and ASD, which has recently been referred to as profound autism [17], is associated with even higher rates of psychotropic drug prescription [4]. In a multicenter Italian study, psychotropic drugs were prescribed to over half of adults with ASD, while only 15% had a diagnosed psychiatric disorder, and appropriateness of treatment was confirmed in barely 10% of cases [14]. Such findings highlight how diagnostic overshadowing and limited specialist training contribute to pharmacological practices that are poorly aligned with evidence-based criteria. As emphasized by Bertelli [18], the rationale for psychotropic prescribing in people with ID/ASD should rest on interdisciplinarity formulation, precision, personalization, and participation, ensuring that medication use follows comprehensive psychiatric assessment rather than compensating for its absence.

Within the Italian National Health Service, healthcare is publicly funded and, with few exceptions, people with ID access the same primary and specialist services as the general population. Recent work describing the Italian context has highlighted several critical issues: the scarcity of specialized services for adults with ID, the lack of robust prevalence data on adults with ID, and the tendency for disability policies and resources to target “disability” as a broad category without specific provision for ID or autism-related needs [19]. In this fragmented framework, mental health care for adults with ID is often delivered within generic psychiatric services, which may not always be adapted to their communication, cognitive, and support needs. Against this background, locally developed specialist services, such as the facility described in the present study, represent progressive attempts to provide more tailored, integrated responses for adults with ID and co-occurring psychiatric disorders.

These principles underpin the need for specialized services capable of integrating diagnostic expertise with individualized treatment planning. Recognizing this critical knowledge gap and the limitations of existing service models, the present study examines the diagnostic and treatment outcomes achieved through specialized psychiatric assessment in a community-based sample of adults with ID, both with and without co-occurring ASD. Therefore, this study aims to confirm the hypothesis that precision diagnosis and targeted treatment, facilitated by specialist assessment, can: (i) reduce the number of psychopharmacological prescriptions; (ii) reduce the burden of side effects; and (iii) improve clinical outcomes in adults with ID and co-occurring psychiatric disorders.

## 2. Materials and Methods

### 2.1. Study Design

This was an observational retrospective study that followed the STROBE (Strengthening the Reporting of Observational Studies in Epidemiology) guidelines for reporting observational studies [13].

### 2.2. Study Setting

The retrospective data for this study were obtained from clinical records of individuals with ID, with or without ASD, who received psychiatric care at the facility Transition Service, within the Azienda Sanitaria Friuli Occidentale (ASFO) Health Authority, Italy. The service operates across the entire ASFO catchment area, serving a population of 310,981 inhabitants, and it was established in 2021 as part of a regional initiative for social and health integration for people with cognitive disabilities. The service provides comprehensive support to young adults with ID, both with and without ASD, and their families during the transition from infant to adult-oriented disability services. It also serves older adults with neurodevelopmental disabilities, who are facing critical life transitions, including changes in family circumstances, housing arrangements, health status, and levels of functioning. These transitional phases often represent periods of increased vulnerability, during which the risk of psychiatric decompensation and service discontinuity may rise [20]. Approximately 200 new patients are referred to the service annually. Individuals typically receive monthly psychiatric evaluations, with additional follow-up appointments scheduled according to clinical need. The Transition Service provides specialized psychiatric services tailored to the unique needs of adults with ID and ASD, addressing a critical gap in community-based mental health care. The psychiatrists’ team comprises three specialists (MBa, MBe, MR), who employ a multimodal assessment approach, combining direct observation, family and carer interviews, and the administration of psychodiagnostic tools validated for this specific population, including instruments from the SPAIDD battery [21]. Psychiatric assessment is initiated following multidisciplinary review if concerns arise about psychiatric comorbidity or if a patient with an existing psychiatric diagnosis requires further evaluation of their psychopharmacological treatment. Thus, the study sample reflects adults with ID referred to a newly established specialist service within the Italian National Health Service, rather than a narrowly selected research cohort, and is therefore representative of routine clinical activity in this setting. Within the Transition Service, psychiatric care routinely combines pharmacological treatment with non-pharmacological interventions, including psychoeducational, behavioral, and supportive strategies for patients and caregivers. However, because non-pharmacological interventions were not recorded in a standardized manner across all cases, the present retrospective study focused on pharmacological management and related outcomes.

### 2.3. Study Cohort

The study cohort comprised adults with ID, with or without ASD, who underwent specialized psychiatric assessment at the Transition Service. For this study, we considered all individuals who attended the service between January 2023 and January 2024 and met the predefined inclusion criteria. From this eligible pool (*n* = 84), one out of every three patients was randomly selected for analysis.

Inclusion criteria:

- Adults (aged 18 or older) with a documented diagnosis of ID, with or without ASD;
- Patients requiring evaluation of a psychopharmacological intervention or modification of their current treatment;
- Patients who screened positive for psychopathology and presented with higher intensity and pervasiveness of symptoms or challenging behaviors, as determined by both the SPAIDD-G (Systematic Psychopathological Assessment for Persons with Intellectual and Developmental Disabilities-General) scale [21] and clinical impression.

Exclusion criteria:

- Individuals who did not receive a follow-up assessment after the initial visit, either because the psychiatric team determined that further psychiatric evaluation was not indicated at that time (for instance, when referral to other types of care, such as assessment of physical comorbidities, was considered more appropriate), or in cases where the patient withdrew from the assessment program.

### 2.4. Data Source

Information gathered during the assessment process and documented in the individual’s clinical record served as the primary data source. Data were collected from clinical files and clinicians’ notes. Data extraction was performed by three investigators (MBa, MR, MBe), using a standardized data collection form developed specifically for this study. A random sample of 10% of records was independently reviewed by a second investigator to ensure data extraction reliability.

### 2.5. Collected Data

- Socio-demographic characteristics: age (years) and sex (male/female)
- Neurodevelopmental disorders:
  - Presence of ID;
  - Severity of ID (mild, moderate, severe, profound);
  - Presence of ASD;
  - Presence of profound ASD [17].
- Organic and neurological comorbidities:
  - Presence of organic comorbidities;
  - Presence of neurological comorbidities;
  - Presence of epilepsy (yes/no).
- Psychopathological comorbidities: psychiatric diagnoses were recorded before and after specialist assessment within the service. Diagnoses were made according to DM-ID2 criteria [15] and classified into broader categories, utilizing an instrumental battery including the SPAIDD-G, a general screening tool for psychopathology [21] and diagnostic area-specific tools: the SPAIDD-P (Systematic Psychopathological Assessment for Persons with Intellectual and Developmental Disabilities—Psychotic Disorders) [22] for the diagnosis of psychotic disorders and the SPAIDD-M (Systematic Psychopathological Assessment for Persons with Intellectual and Developmental Disabilities—Mood Disorders) [23,24] for identification of mood disorders.
- Psychotropic medications use: Psychotropic medications prescribed before and after specialist assessment (reported by class).
- Psychotropic medications’ side effects: Presence of side effects related to psychopharmacological therapy before and after specialist assessment.
- Clinical outcomes: They were systematically assessed by clinicians using a structured rating form developed as part of the SPAIDD psychodiagnostic battery [21], specifically adapted for follow-up assessment, the Systematic Psychopathological Assessment for Persons with Intellectual and Developmental Disabilities—Follow-Up (SPAIDD-FU). This tool, currently used internally within the facility to monitor patient progress, comprises a checklist of 25 psychopathological dimensions commonly observed in individuals with ID: aggression, destructiveness, self-harm, psychomotor agitation, somatic complaints, pica, hyperactivity, impulsivity, oppositional behavior, thefts, phobias, stereotypies, apathy, abulia, low mood, elevated mood, sleep disturbances, eating problems, autistic behavior, negativism, obsession, compulsions, delusions, visual and auditory hallucinations. Each dimension is rated for both intensity (0 = Absent, 1 = Mild, 2 = Moderate, 3 = Severe) and frequency (1 = Monthly, 2 = Weekly, 3 = Daily, 4 = Trait/Continuous). The SPAIDD-FU is routinely administered at baseline (first psychiatric contact) and at subsequent follow-ups. For the present study, data were extracted from the SPAIDD-FU assessments completed at baseline and three months after initiation of targeted treatment informed by the specialist evaluation. This timeframe was selected to capture short-term clinical changes resulting from individualized diagnostic clarification and treatment optimization.

### 2.6. Statistical Analysis

Categorical variables were presented as frequencies and/or percentages, continuous variables as means and standard deviation (SD). Non-parametric analyses were preferred for paired, pre-post, comparisons (Wilcoxon test, W; McNemar test with continuity correction, χ^2^). We also examined differences between independent groups (i.e., sex, level of ID and ASD, and presence of comorbidities), again using non-parametric between-group analyses (Mann–Whitney test, U; Fisher exact test). Additionally, Spearman correlations were used (ρ). Statistical significance was set at α = 0.05 (two-tailed). This study was intended to be entirely preliminary and exploratory in nature, particularly in light of the limited sample size that discourages a formal correction for multiple independent comparisons. For this reason, we explicitly acknowledged these limitations and chose to report statistically significant differences together with a clear indication of their corresponding significance levels (i.e., 0.05, 0.01, 0.001). Analyses were conducted in R-4.5.1 (www.R-project.org).

### 2.7. Ethical Considerations

The study was conducted in accordance with the Declaration of Helsinki. Given the retrospective nature of the study using anonymized clinical data, informed consent was waived. All data were de-identified prior to analysis to ensure patient confidentiality.

## 3. Results

### 3.1. Demographic and Clinical Characteristics

Table 1 details the demographic and clinical characteristics of the study sample at enrollment. A total of 28 patients were initially selected; however, three were excluded from the analyses as they did not meet the inclusion criteria due to discontinuation of the assessment process. Specifically, one family moved to another region, one family disagreed with the diagnosis made by the team, and one patient required prolonged hospitalization for management of organic comorbidities. Therefore, the final sample comprised 25 individuals, all with a diagnosis of ID. Participants diagnosed with ASD were significantly younger (26.5 ± 9.74 vs. 38.6 ± 11.26 years old; U = 128.5, *p* = 0.006) and more often males (69.2% vs. 30.8% females; *p* = 0.047) than others. The same was true for participants with profound autism (age: U = 123.5, *p* = 0.012; sex: *p* = 0.047). Also, a statistically significant association was observed between level of ID and epilepsy—with epilepsy present only in participants with severe or profound ID (*p* = 0.015).

### 3.2. Changes in Psychiatric Diagnoses Following Specialized Assessment

At baseline, 32% of the sample had at least one psychiatric diagnosis, which increased to 96% after the assessment (from 8 to 24 individuals; χ^2^ = 12.5, *p* < 0.001). In particular, 17 participants who initially had no diagnosis received one, while only one previously diagnosed participant was no longer diagnosable. The mean number of diagnoses per patient also significantly increased (W = 29.5, *p* < 0.001). Diagnoses showing statistically significant changes were bipolar disorders (χ^2^ = 4.0, *p* = 0.046), depressive disorders (χ^2^ = 6.1, *p* = 0.013), and personality disorders (χ^2^ = 4.2, *p* = 0.041). Interestingly, while emerging as significant comorbidities following specialist assessment, depressive and personality disorders had not been diagnosed at baseline in any participant. As reported in Table 2, the overall diagnostic profile of the sample was markedly different after the specialistic assessment. These diagnostic transitions are also illustrated in the Sankey diagram provided in Figure S1. Moreover, after specialized assessment, a personality disorder was assigned only to females (*p* = 0.015) and to participants without ASD (*p* = 0.005). Also, bipolar disorder was more often diagnosed in patients with profound ASD compared to others (63.6% vs. 14.3%; *p* = 0.017).

### 3.3. Changes in Psychopharmacological Treatments and Side Effects Following Specialized Assessment

Before referral to the Transition Service, psychotropic medications were generally prescribed and managed within generic services, most commonly by community psychiatrists and, in some cases, by other physicians such as neurologists or general practitioners. Within the Transition Service, all subsequent changes to psychotropic regimens were made by the specialist psychiatric team. At baseline, 92% of the sample (*n* = 23/25) was taking at least one psychotropic medication, and 60% of patients (*n* = 15/25) had a prescription in the absence of any formal diagnosis of psychopathological co-occurrence. At follow-up, all patients were taking at least one psychotropic medication, but only 4% of the sample (*n* = 1/25) had a prescription without a formal psychopathological diagnosis. With regard to specific pharmacological classes, as shown in Table 3, after specialist assessment there was a significant change in the proportion of patients prescribed with first-generation antipsychotics, which dropped from 36% (*n* = 9/25) to 8% (*n* = 2/25) (χ^2^ = 5.1, *p* = 0.023) and antidepressants, with an increase from 12% (*n* = 3/25) to 52.0% (*n* = 13/25) (χ^2^ = 8.1, *p* = 0.004). Among the patients who received a new prescription of antidepressants at follow-up, four patients had received a diagnosis of depressive disorder, three had been diagnosed with bipolar, four patients received a diagnosis of anxiety disorder and one of impulse control disorder.

The use of second-generation antipsychotics, instead, did not show statistically significant differences, with 76% (*n* = 19/25) of the sample receiving these medications both before and after the intervention (even though four participants changed their prescription, with two starting a new medication and two discontinuing the previous one). Given the overlap in antipsychotic use, combination treatment with both first- and second-generation agents was analyzed separately. As presented in Table 3, 28% of patients (*n* = 7/25) were receiving both classes at baseline, whereas only 8% (*n* = 2/25) remained on combination therapy after the assessment. The follow-up evaluation resulted in six discontinuations and one new initiation, thereby highlighting the reduction in patients receiving both antipsychotic classes. Also, the rate of patients prescribed with any psychotropic medication and the mean number of drugs prescribed per patient were not statistically different between evaluations. As shown in Table 4, the total number of side effects per patient significantly changed following the specialist assessment (from a mean of 1.6 to 0.5 per participant; U = 153.0, *p* < 0.001), with the prevalence of any side effect decreasing significantly, from 72% (*n* = 18/25) to 44% (*n* = 11/25) (χ^2^ = 5.1, *p* = 0.023). Specifically, significant differences were observed in the proportion of patients with increased prolactin levels (χ^2^ = 4.2, *p* = 0.041) and psychomotor retardation (χ^2^ = 4.2, *p* = 0.041).

At baseline, patients with organic comorbidities were taking a higher number of medications than others (on average: 2.9 ± 1.49 vs. 1.7 ± 1.42; U = 39.5, *p* = 0.048), with more frequent use of mood stabilizers (53.3% vs. 10.0%; *p* = 0.040) and sedatives (66.7% vs. 20.0%; *p* = 0.041). Instead, participants with neurological comorbidities were prescribed more 1st-generation antipsychotics (66.7% vs. 18.8%; *p* = 0.031), despite the fact that only two of them had a formal psychiatric diagnosis, specifically bipolar disorder in one case and dementia in the other. These patients also experienced more side effects at both baseline (2.3 ± 1.32 vs. 1.12 ± 1.20; U = 33.5, *p* = 0.026) and follow-up (1.0 ± 0.71 vs. 0.3 ± 0.45; U = 30.0, *p* = 0.008). Specifically, they experienced more extrapyramidal symptoms (*p* = 0.014) at baseline and more sedation (*p* = 0.002) at follow-up. Considering patients with epilepsy, they assumed more frequently mood stabilizers (80.0% vs. 25.0%; *p* = 0.040) and 1st-generation antipsychotics (80.0% vs. 25.0%; *p* = 0.040). Finally, after specialized assessment, patients with antidepressants prescription were younger than those without (27.2 ± 11.26 vs. 37.8 ± 10.61 years old; U = 119.5, *p* = 0.026).

### 3.4. Changes in Symptoms’ Frequency and Intensity

Table 5 presents the changes in intensity and frequency of psychopathological symptoms and challenging behaviors as measured by the SPAIDD-FU. Following the specialist assessment and targeted treatment, statistically significant mean reductions in symptom intensity were observed (U = 276.0, *p* < 0.001). As for specific items in SPAIDD-FU, a statistically significant decrease was observed for: aggression (U = 120.0, *p* < 0.001), psychomotor agitation (U = 120.0, *p* < 0.001), hyperactivity (U = 21.0, *p* = 0.031), impulsivity (U = 66.0, *p* = 0.002), oppositional behavior (U = 55.0, *p* = 0.003), stereotypies (U = 15.0, *p* = 0.048), apathy (U = 36.0, *p* = 0.010), abulia (U = 28.0, *p* = 0.018), low mood (U = 45.0, *p* = 0.007), elevated mood (U = 28.0, *p* = 0.015), sleep disturbances (U = 55.0, *p* = 0.004), obsessions (U = 28.0, *p* = 0.011), and compulsions (U = 28.0, *p* = 0.015). Similarly, a significant reduction resulted in mean symptom frequency (U = 231.0, *p* < 0.001) for: aggression (U = 120.0, *p* < 0.001), psychomotor agitation (U = 28.0, *p* = 0.018), impulsivity (U = 21.0, *p* = 0.034), low mood (U = 28.0, *p* = 0.019), and compulsions (U = 15.0, *p* = 0.037) (Table 5).

SPAIDD-FU mean intensity was negatively correlated with age of participants at both baseline (ρ = −0.628, *p* < 0.001) and FU (ρ = −0.512, *p* = 0.009). Mean frequency was similarly associated with age at baseline (ρ = −0.465, *p* = 0.019), but only a trend was observed at FU (ρ = −0.354, *p* = 0.082). Mean intensity and frequency of symptoms were also higher for patients with ASD or profound ASD at both baseline (all with *p* ≥ 0.002) and FU (all with *p* ≥ 0.004).

After specialized assessment, reduction in both mean symptoms’ intensity and frequency at SPAIDD-FU was lower in patients with organic comorbidities than in those without (intensity: −0.4 ± 0.20 vs. −0.2 ± 0.11; U = 33.0, *p* = 0.021; frequency: −0.1 ± 0.12 vs. −0.3 ± 0.18; U = 34.5, *p* = 0.026).

## 4. Discussion

The present study provides evidence that specialized psychiatric assessment in adults with intellectual disability (ID) and co-occurring psychiatric disorders yields substantial improvements in diagnostic accuracy, medication management, and clinical outcomes within community-based settings. These findings are particularly significant considering both the heightened psychopathological vulnerability observed in this population [1] and the well-documented barriers to accessing specialized mental healthcare [2].

While specialist psychiatric services for adults with intellectual disabilities are not universally available within the Italian national health system [19], innovative services can be implemented at the initiative of individual administrations, as demonstrated by the service that is the subject of this investigation.

The comprehensive assessment carried out by trained psychiatrist allowed to frame the challenging behaviors within defined psychopathological conditions and to refine existing psychiatric diagnoses. The substantial increase in the proportion of patients with at least one psychiatric diagnosis—from 32% (*n* = 8/25) at baseline to 96% (*n* = 24/25) after specialized assessment—is therefore best interpreted as improved recognition of co-occurring mental disorders in a previously underdiagnosed group, rather than overdiagnosis. This finding aligns with recent European guidelines emphasizing the need for adapted assessment methods to reduce diagnostic overshadowing—the tendency to attribute psychiatric symptoms to the intellectual disability itself [25]. The emergence of depressive disorders (0% to 32%, *n* = 0–8/25) and personality disorders (0% to 24%, *n* = 0–8/25) is particularly noteworthy, as these conditions are frequently underdiagnosed in ID populations despite their prevalence in general psychiatric settings [26]. The substantial increases in bipolar disorders (from 8% to 36%, *n* = 2–9/25), anxiety disorders (from 4% to 24%, *n* = 1–8/25), and impulse control disorders (from 0% to 16%, *n* = 0–4/25) deserve particular attention as they represent conditions that are often misinterpreted in individuals with ID. Bipolar disorder symptoms, particularly manic episodes, may be mistakenly attributed to behavioral problems rather than recognized as manifestations of a treatable psychiatric condition [27]. Similarly, anxiety presentations in individuals with ID are frequently overlooked or mischaracterized as part of the developmental disability, despite anxiety disorders being among the most prevalent psychiatric comorbidities in this population [28]. Impulse control disorders, which were entirely undiagnosed at baseline, likely reflect the specialized clinicians’ ability to distinguish pathological impulsivity from the behavioral disinhibition that can accompany ID [29]. These diagnostic improvements have direct therapeutic implications, as each condition requires specific pharmacological and psychosocial interventions that differ substantially from generic behavioral management approaches. These data also indicate that a specialist approach provide a more comprehensive and nuanced understanding of the phenomenology of mental distress in individuals with intellectual disability—leading to diagnostic profiles that more closely align with those observed in the general psychiatric outpatient population [4].

In line with previous research [3], approximately half of the sample was prescribed psychotropic medications without a formal psychiatric diagnosis prior to the specialized assessment, frequently as a means of behavior management rather than targeted treatment of a defined psychiatric condition [14,30]. Our findings, conversely, demonstrate that specialized psychiatric assessment substantially increases diagnostic accuracy, thereby allowing a shift from non-specific pharmacotherapy toward rational, diagnosis-driven prescriptions. This improvement reflects the implementation of what Bertelli [18] described as an interdisciplinary, precision-based and participatory model of psychopharmacological care, in which treatment decisions are continuously reviewed and integrated with behavioral and psychosocial approaches. Consistent with these theoretical and ethical principles, our data confirm that systematic reassessment and diagnostic refinement can effectively reduce the use of high-risk medications—particularly first-generation antipsychotics—and minimize adverse effects, without increasing overall pharmacological burden.

The diagnostic refinement prompted a change in psychopharmacological treatment in the vast majority of cases (96%, *n* = 24/25), with several consistent patterns emerging. The significant reduction in first-generation antipsychotic prescribing—and, to a lesser extent, of sedative and anticholinergic drugs—and concurrent increase in antidepressant use reflects a shift toward evidence-based prescribing. This pattern mirrors broader trends documented in recent population studies, where antidepressant prescribing for adults with ID has increased markedly, outpacing rises in the general population [16,31]. However, our findings extend beyond population trends by demonstrating that this shift can be achieved through specialist assessment while maintaining stable the overall psychotropic burden. The persistence of second-generation antipsychotic prescribing in 76% of the sample (*n* = 19/25) likely reflects their continued role in managing severe behavioral problems, consistent with meta-analytic evidence showing modest but significant efficacy for acute challenging behaviors [32]. Notably, despite the mean number of drugs prescribed per patient remained substantially stable, the burden of side effects significantly decreased.

The significant decrease in total side effects per patient is clinically meaningful and aligns with treatment optimization following specialist assessment. Because more accurate diagnoses enabled more appropriate prescribing, the decrease in side effects reflects an expected therapeutic benefit rather than a chance occurrence. The specific reductions in prolactin elevation and psychomotor retardation likely reflect the decreased use of first-generation antipsychotics, which carry higher risks for these side effects. It is worth noting that the significant reduction in the prescription of first-generation antipsychotics, along with sedatives and anticholinergics, did not make the behavior any worse, but instead made a significant improvement in the medication-related side effects.

The significant reductions in both symptom intensity and frequency across multiple domains (aggression, psychomotor agitation, mood disturbances, sleep problems, and obsessive-compulsive symptoms) demonstrate the clinical utility of specialized assessment. Notably, our finding that symptom severity was negatively correlated with age—with younger participants presenting more severe symptomatology—aligns with the well-documented challenges of the transition period from childhood to adulthood in neurodevelopmental disabilities [33,34]. Similarly, the more severe clinical presentations observed in individuals with profound ASD underscore the critical need for tailored approaches that account for this specific phenotype [17]. These findings underscore the importance of developing stratified approaches to psychiatric care that consider both developmental stage and autism severity when designing interventions for adults with ID. The finding that symptom reduction was lower in patients with organic comorbidities aligns with the complexity of managing psychiatric symptoms in the context of neurological conditions, an area requiring further research attention [35].

## 5. Conclusions

The single-center design and modest sample size of this study constrain the reliability of the observed differences. These findings should thus be generalized only with caution, indicating the need for further investigation. A further limitation is that data on non-pharmacological interventions (e.g., behavioral, psychoeducational, or psychosocial approaches) were not collected. As a result, the relative contribution of these interventions to the observed clinical improvements could not be assessed, and our findings mainly reflect the pharmacological component of treatment. Future research should examine the sustainability of these improvements over longer follow-up periods. Additionally, the development of standardized protocols for psychiatric assessment in adults with ID could facilitate broader implementation of these approaches, considering the fact that it may be challenging to make an accurate psychiatric diagnosis in this population, particularly among those with severe and profound ID, because of communication problems and potential different manifestations of psychiatric symptoms in this population [36].

## Figures and Tables

**Table 1:** Demographic and clinical characteristics at baseline.

Total Sample (N = 25)	M ± SD or *n* (%)
Age, years	32.3 ± 11.99
Sex, females	13 (52%)
Intellectual Disability:	
- Mild	1 (4%)
- Moderate	12 (48%)
- Severe	11 (44%)
- Profound	1 (4%)
Autism Spectrum Disorder	13 (52%)
Profound Autism	11 (44%)
Genetic comorbidities	9 (36%)
Organic comorbidity	15 (60%)
Neurological comorbidity	9 (36%)
Epilepsy	5 (20%)

Footnotes: M: Mean; N: total number of observations; *n*: Number of observations in group; SD: Standard deviation.

**Table 2:** Changes in psychopathological diagnoses before (BL) and after (FU) specialized assessment.

Psychopathological Diagnoses	BL, *n* (%)	FU, *n* (%)	Changes at FU	
Neurodevelopmental disorders:				
- Tic	1 (4%)	-	New: 0, Lost: 1	
- ADHD	1 (4%)	2 (8%)	New: 2, Lost: 1	
Schizophrenia spectrum disorders	3 (12%)	2 (8%)	New: 1, Lost: 2	
Bipolar disorders	2 (8%)	9 (36%)	New: 8, Lost: 1	*
Depressive disorders	-	8 (32%)	New: 8, Lost: 0	*
Anxiety disorders	1 (4%)	6 (24%)	New: 6, Lost: 1	
Obsessive–compulsive disorder	2 (8%)	1 (4%)	New: 1, Lost: 2	
Somatic symptom disorders	-	2 (8%)	New: 2, Lost: 0	
Sleep–wake disorders	-	1 (4%)	New: 1, Lost: 0	
Impulse control disorders	-	4 (16%)	New: 4, Lost: 0	
Neurocognitive disorders	2 (8%)	2 (8%)	New: 2, Lost: 2	
Personality disorders	-	6 (24%)	New: 6, Lost: 0	*
Any diagnoses	8 (32%)	24 (96%)	New: 17, Lost: 1	***
Total number of diagnoses, M ± SD	0.48 ± 0.918	1.72 ± 0.678	Δ(FU-BL): +1.24 ± 1.200	***

Footnotes: ADHD: Attention-Deficit/Hyperactivity Disorder; BL: Baseline (before specialized assessment); FU: Follow-Up (after specialized assessment); M: Mean; *n*: Number of observations in group; SD: Standard deviation. * *p* < 0.050, *** *p* < 0.001.

**Table 3:** Changes in psychotropic prescription before (BL) and after (FU) specialist assessment.

Pharmacological Class	BL, *n* (%)	FU, *n* (%)	Changes at FU	
1st-generation antipsychotics	9 (36%)	2 (8%)	New: 0, Discontinued: 7	*
2nd-generation antipsychotics	19 (76%)	19 (76%)	New: 2, Discontinued: 2	
1st- + 2nd-generation antipsychotics	7 (28%)	2 (8%)	New: 1, Discontinued: 6	
Antidepressants	3 (12%)	13 (52%)	New: 10, Discontinued: 0	**
Mood stabilizers—Antiepileptics	9 (36%)	11 (44%)	New: 3, Discontinued: 1	
Sedatives	12 (48%)	7 (28%)	New: 1, Discontinued: 6	
Anticholinergics	3 (12%)	0	New: 0, Discontinued: 3	
Pro-cognitive drugs	0	1 (4%)	New: 1, Discontinued: 0	
Psychostimulants	0	1 (4%)	New: 1, Discontinued: 0	
Any drug	23 (92%)	25 (100%)	New: 2, Discontinued: 0	
Total number of drugs, M ± SD	2.44 ± 1.557	2.48 ± 1.418	Δ(FU-BL): +0.04 ± 1.172	

Footnotes: BL: Baseline (before specialized assessment); FU: Follow-Up (after specialized assessment); M: Mean; *n*: Number of observations in group; SD: Standard deviation. * *p* < 0.050, ** *p* < 0.01.

**Table 4:** Changes in side effects related to psychotropic medication before (BL) and after (FU) specialist assessment.

Side Effect	BL, *n* (%)	FU, *n* (%)	Changes at FU	
QTc prolongation	2 (8.7%)	0	New: 0, Remitted: 2	
Weight gain	7 (30.4%)	4 (17.4%)	New: 0, Remitted: 3	
Increased prolactin	6 (26.1%)	0	New: 0, Remitted: 6	*
Drooling	2 (8.7%)	1 (4.3%)	New: 0, Remitted: 1	
Psychomotor retardation	11 (47.8%)	5 (21.7%)	New: 0, Remitted: 6	*
Extrapyramidal symptoms	4 (17.4%)	1 (4.3%)	New: 0, Remitted: 3	
Sedation	6 (26.1%)	1 (4.3%)	New: 0, Remitted: 5	
Irritability	1 (4.3%)	1 (4.3%)	New: 1, Remitted: 1	
Any side effect	18 (72%)	11 (44%)	New: 0, Remitted: 7	*
Total *n* of side effects, M ± SD	1.56 ± 1.356	0.52 ± 0.653	Δ(FU-BL): −1.04 ± 0.935	***

Footnotes: BL: Baseline (before specialized assessment); FU: Follow-Up (after specialized assessment); M: Mean; *n*: Number of observations in group; SD: Standard deviation. * *p* < 0.050, *** *p* < 0.001.

**Table 5:** Change in intensity and frequency of psychopathological symptoms and challenging behaviors before (BL) and after (FU) specialist assessment.

Symptom/Behavior	Intensity, M ± SD			Frequency, M ± SD		
	BL	FU		BL	FU	
1. Aggression	1.68 ± 1.030	1.04 ± 0.790	***	1.92 ± 1.115	1.24 ± 0.879	***
2. Destructiveness	0.16 ± 0.473	0.08 ± 0.277		0.24 ± 0.723	0.16 ± 0.554	
3. Self-harm	0.56 ± 1.003	0.44 ± 0.870		0.68 ± 1.145	0.44 ± 0.870	
4. Psychomotor agitation	1.68 ± 1.069	1.00 ± 0.764	***	1.64 ± 1.114	1.28 ± 1.021	**
5. Somatic complaints	0.48 ± 0.918	0.32 ± 0.627		0.56 ± 1.003	0.36 ± 0.700	
6. Pica	-	-		-	-	
7. Hyperactivity	0.88 ± 1.166	0.56 ± 0.821	*	1.08 ± 1.441	0.84 ± 1.248	
8. Impulsivity	1.40 ± 1.041	0.92 ± 0.909	**	1.92 ± 1.412	1.52 ± 1.418	*
9. Oppositional behavior	1.28 ± 1.061	0.84 ± 0.800	**	1.60 ± 1.291	1.48 ± 1.358	
10. Thefts	-	-		-	-	
11. Phobias	0.56 ± 0.961	0.28 ± 0.614		0.64 ± 1.075	0.40 ± 0.866	
12. Stereotypies	1.00 ± 1.155	0.76 ± 0.926	*	1.64 ± 1.777	1.64 ± 1.777	
13. Apathy	0.84 ± 1.106	0.44 ± 0.712	*	1.08 ± 1.412	1.00 ± 1.414	
14. Abulia	0.76 ± 1.052	0.40 ± 0.707	*	1.08 ± 1.412	0.88 ± 1.364	
15. Low mood	0.88 ± 1.054	0.32 ± 0.627	**	1.08 ± 1.352	0.48 ± 1.005	*
16. Elevated mood	0.72 ± 1.021	0.40 ± 0.645	*	0.64 ± 0.952	0.48 ± 0.823	
17. Sleep disturbances	1.00 ± 1.041	0.52 ± 0.653	**	1.00 ± 1.080	0.72 ± 0.936	
18. Eating problems	0.32 ± 0.748	0.32 ± 0.690		0.44 ± 1.044	0.52 ± 1.085	
19. Autistic behavior	1.28 ± 1.208	1.24 ± 1.234		2.36 ± 1.977	2.24 ± 2.026	
20. Negativism	0.16 ± 0.554	0.08 ± 0.400		0.24 ± 0.831	0.12 ± 0.600	
21. Obsessions	0.88 ± 1.054	0.60 ± 0.816	*	1.20 ± 1.414	1.12 ± 1.333	
22. Compulsions	0.96 ± 1.098	0.64 ± 0.757	*	1.36 ± 1.469	1.16 ± 1.281	*
23. Delusions	0.08 ± 0.400	0.04 ± 0.200		0.16 ± 0.624	0.16 ± 0.624	
24. Visual hallucinations	0.16 ± 0.554	0.08 ± 0.400		0.24 ± 0.831	0.12 ± 0.600	
25. Auditory hallucinations	0.24 ± 0.663	0.12 ± 0.440		0.36 ± 0.995	0.20 ± 0.707	
Overall (mean)	0.72 ± 0.261	0.46 ± 0.263	***	0.93 ± 0.325	0.74 ± 0.354	***

Footnotes: BL: Baseline (before specialized assessment); FU: Follow-Up (after specialized assessment); M: Mean; SD: Standard deviation. * *p* < 0.050, ** *p* < 0.01, *** *p* < 0.001.

## Data Availability

The data presented in this study are available on request from the corresponding author due to restrictions related to privacy and ethical reasons.

## Supplementary Materials

The following supporting information can be downloaded at: https://www.mdpi.com/article/10.3390/jcm15020489/s1. Figure S1: Changes in psychopathological diagnoses before and after specialized assessment.

## Abbreviations

The following abbreviations are used in this manuscript:

ID	Intellectual disability
ASD	Autism Spectrum Disorder
ASFO	Azienda Sanitaria Friuli Occidentale
SPAIDD	Systematic Psychopathological Assessment for Persons with Intellectual and Developmental Disabilities
DM-ID2	Diagnostic Manual—Intellectual Disability, Second Edition
FGAP	First-Generation Antipsychotics
SGAP	Second-Generation Antipsychotics
BL	Baseline
FU	Follow-up
ADHD	Attention Deficit and Hyperactivity Disorder
M	Mean
SD	Standard Deviation
