# Supplementary material for: Effect of Specialized Psychiatric Assessment and Precision Diagnosis on Pharmacotherapy in Adults with Intellectual Disability
Source: J Clin Med. 2026 Jan 8;15(2):489. doi: 10.3390/jcm15020489 (PMC12842143; doi:10.3390/jcm15020489)
Supplement: Supplementary file 1 [file jcm-15-00489-s001.zip › jcm-4050445-supplementary.pdf]

Figure S1. Changes in psychopathological diagnoses before and after specialized assessment.

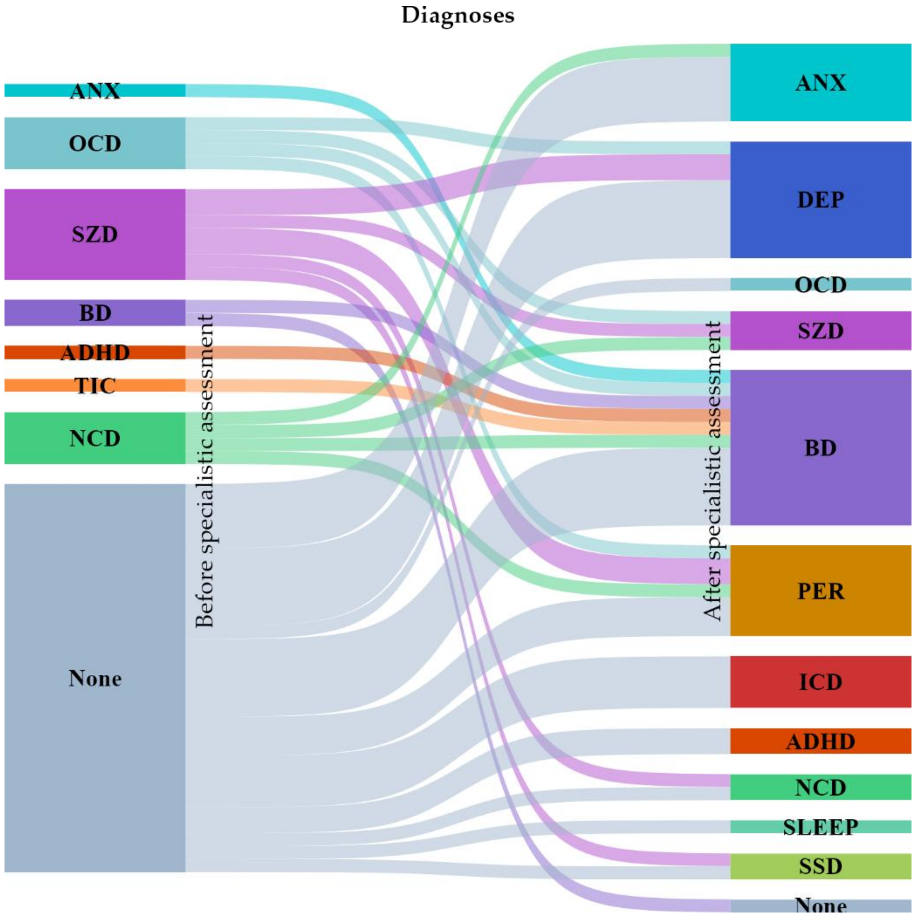

Footnotes: **ADHD:** Attention-Deficit/Hyperactivity Disorder; **ANX:** Anxiety Disorders; **BD:** Bipolar Disorders; **DEP:** Depressive Disorders; **ICD:** Impulse Control Disorders; **NCD:** Neurocognitive Disorders; **OCD:** Obsessive-Compulsive Disorder; **PD:** Personality Disorders; **SLEEP:** Sleep-Wake Disorders; **SSD:** Somatic Symptom Disorders; **SZD:** Schizophrenia Spectrum Disorders; **TIC:** Tic Disorders.
